# A Case of a Perforated Duodenal Ulcer in the Context of Acute Tubular Necrosis and Urosepsis

**DOI:** 10.7759/cureus.92482

**Published:** 2025-09-16

**Authors:** Michael O Roth, Jae Hwan Choi, Saar Peles, Aron S Mcguirt

**Affiliations:** 1 Internal Medicine, University of Central Florida College of Medicine, Orlando, USA; 2 General Surgery, University of South Florida, Tampa, USA; 3 Gastrointestinal Oncology, University of Central Florida College of Medicine, Orlando, USA; 4 General Surgery, Bay Pines VA Healthcare System, St. Petersburg, USA

**Keywords:** acute kidney injury, exploratory laparotomy, perforated duodenal ulcer, stress-induced ulcer, urosepsis

## Abstract

Duodenal ulcer perforation is a life-threatening complication of peptic ulcer disease (PUD), often manifesting with acute abdominal symptoms. However, PUD itself can be asymptomatic, potentially delaying diagnosis, particularly in individuals without recognized pre-existing gastrointestinal conditions. While *Helicobacter pylori* infection and the use of nonsteroidal anti-inflammatory drugs are well-established primary risk factors for PUD, the condition can also develop in the absence of these common triggers or due to other less apparent causes. This report details the case of an 80-year-old male with chronic kidney disease stage 3A and a recent complicated urinary tract infection (extended-spectrum beta-lactamase-producing *Escherichia coli*) who presented with confusion and increased urinary frequency. His initial evaluation revealed acute kidney injury (AKI) secondary to severe dehydration and metabolic acidosis, which then progressed to urosepsis and ischemic acute tubular necrosis. Unexpectedly, during his hospital admission, he developed a sudden anterior duodenal ulcer perforation. His condition was managed successfully with an emergency exploratory laparotomy and surgical repair, followed by postoperative hemodialysis, leading to a full recovery without complications from the ulcer repair. As this patient lacked typical PUD risk factors, the ulcer was likely idiopathic in origin, with its acute presentation likely exacerbated by AKI-associated uremia, metabolic acidosis, and the physiological stress of his critical illness. This case underscores the importance of recognizing stress-related risk factors for peptic ulcer formation in critically ill patients with AKI, as prophylactic measures recommended to prevent stress ulcer bleeding may also help reduce the risk of life-threatening complications such as perforation.

## Introduction

Perforation of an ulcer in the stomach or duodenum is a life-threatening complication of an often-insidious underlying case of peptic ulcer disease (PUD), presenting with sudden-onset pain in the upper abdomen as well as a distended abdomen and pneumoperitoneum on imaging [1]. These ulcers arise from damage to the protective mucosal lining of the stomach and duodenum, rendering the underlying tissue vulnerable to gastric acid, with the most prevalent risk factors being *Helicobacter pylori* infection as well as nonsteroidal anti-inflammatory drug (NSAID) and aspirin use [1]. However, at least 20% of PUD cases are noted to be negative for *H. pylori*, NSAID use, or aspirin use, which is termed idiopathic PUD [2]. Despite having a poorly understood clinical course due to the heterogeneity of the patient populations, idiopathic peptic ulcers are associated with higher risk of complications such as perforation or hemorrhage, longer-lasting ulceration, a greater likelihood of presenting as multiple and larger ulcers, more limited response to therapy with proton-pump inhibitors, more severe symptoms of dyspepsia, and higher rate of recurrence and rate of mortality [2]. Idiopathic PUD is a diagnosis of exclusion, as it is critical to rule out rare systemic diseases with effects on the upper gastrointestinal tract, such as sarcoidosis or vasculitis, as well as causes of hyperacidity of the stomach, such as Zollinger-Ellison syndrome [2]. Although it is difficult to elucidate the cause of idiopathic PUD on a patient-by-patient basis, these ulcers are often seen in patients with higher levels of physiological stress or those with life-threatening systemic conditions, both of which may be contributing to ulcer perforation in a patient with severe acute kidney injury (AKI) in addition to the direct effects of uremia in the upper gastrointestinal system and the risk of stress-induced mucosal damage in critically ill patients. Here, we present the case of a perforated duodenal ulcer in an elderly patient with severe AKI and no traditional PUD risk factors, highlighting the diagnostic and management challenges in such scenarios and underscoring the potential role of AKI-related factors in PUD pathogenesis.

## Case presentation

Our patient was an 80-year-old male with a recent history of urinary tract infection (UTI) with extended spectrum beta-lactamase (ESBL)-producing *Escherichia coli* diagnosed 11 days prior that was managed with a seven-day course of nitrofurantoin, as well as a past medical history including essential hypertension, chronic kidney disease (CKD) stage 3A, hypokalemia, multiple bilateral simple renal cysts, non-muscle invasive bladder cancer status post-transurethral resection and Bacillus Calmette-Guerin treatment, benign prostatic hyperplasia with chronically elevated prostate-specific antigen, and hyperlipidemia, who presented to the emergency department (ED) due to altered mental status. His confusion was first noted by his wife, who prompted him to receive further evaluation in the ED. On arrival, he was alert and oriented to person and place but not time. He additionally reported experiencing increasing urinary frequency despite having poor recent oral intake of fluids for the last few days. The patient reported that he had sought care at his primary care provider for treatment of a UTI 11 days prior, as he had been experiencing a two-day history of morning cough, lack of appetite, fatigue, diarrhea, and nighttime fever, stating that he had similar symptoms when he had a UTI in the past. However, he did not report these symptoms when presenting to the ED, stating that he was only experiencing confusion and increased urinary frequency. The patient denied any chest pain, shortness of breath, dysuria, or hematuria. He denied any recent falls or syncope. The patient denied any recent or chronic NSAID use. Vital signs were within normal limits, including a temperature of 98.5°F, heart rate of 99 beats/minute, respiratory rate of 16 breaths/minute, and blood pressure of 121/59 mmHg. Physical examination demonstrated no abnormalities with a regular rate and rhythm on heart auscultation, clear lungs to auscultation bilaterally, a soft and nontender abdomen with no organomegaly, and no costovertebral angle tenderness. Initial laboratory investigations, including a complete blood count and comprehensive metabolic panel, revealed several abnormalities; notably, there was an elevated white blood cell (WBC) count of 17.2 K/µL with 90.5% neutrophils, hyponatremia with a serum sodium of 127 mEq/L, metabolic acidosis with a bicarbonate of 12 mEq/L and an anion gap of 19 mEq/L with lactate of 1.7 mmol/L, and significantly elevated renal markers with a blood urea nitrogen (BUN) of 140 mg/dL and creatinine of 10.64 mg/dL with a known baseline creatinine of 1.37 (Table 1). Initial laboratory investigations also included a hemostatic evaluation, which revealed a normal platelet count, INR, and aPTT, indicating no baseline coagulopathy. Additionally, urinalysis was also indicative of ongoing infection (Table 1). Imaging included a CT of the head without contrast, which showed no acute intracranial pathology, and a negative chest X-ray for acute pathology. Given the patient’s dramatically elevated BUN and creatinine, low serum bicarbonate, high anion gap, and leukocytosis, he was diagnosed with AKI, severe metabolic acidosis, hyponatremia, suspected UTI with possible sepsis, and acute encephalopathy secondary to AKI or infection. A urine culture and sensitivity as well as two sets of blood cultures were performed, and he was admitted to medicine for intravenous (IV) hydration and sodium bicarbonate as well as empiric IV piperacillin-tazobactam.

**Table 1:** Laboratory findings of the patient.

Parameter	Unit	Reference range	Admission (day 1)	Day 3	Preoperative (day 5)	Postoperative (postoperative day 3, before dialysis)
Hematology						
White blood cell count	K/µL	4.0–11.0	17.2	-	19.6	-
Neutrophils	%	40–75	90.5	-	93.7	-
Chemistry panel						
Sodium	mEq/L	135–145	127	136	-	-
Bicarbonate	mEq/L	22–29	12	22	-	-
Anion gap	mEq/L	8–16	19	14	-	-
Blood urea nitrogen	mg/dL	7–20	140	134	138	133
Creatinine	mg/dL	0.7–1.3	10.64	9.91	10.75	-
Phosphate	mg/dL	2.5–4.5	-	7.1	9.3	9.2
Pancreatic enzymes						
Lipase	U/L	<160	-	-	278	-
Amylase	U/L	<100	-	-	327	-
Urinalysis						
Appearance	-	Clear, yellow	Turbid yellow	-	-	-
Leukocyte Esterase	-	Negative	Positive	-	-	-
Nitrites	-	Negative	Negative	-	-	-
White blood cell count	/hpf	0-5	>182	-	-	-
Bacteria	/hpf	None to few	Few	-	-	-
Microbiology						
*Helicobacter pylori* stool antigen	-	Negative	-	-	-	Negative

Over the course of the next four days, the patient’s confusion resolved, and his metabolic acidosis and hyponatremia improved, but his renal function did not show significant improvement with IV fluids, suggesting the development of ischemic acute tubular necrosis, potentially secondary to prolonged dehydration or sepsis. A CT scan of his abdomen and pelvis was performed on day two of his admission, showing multiple bilateral simple renal cysts unchanged from prior imaging with no evidence of hydronephrosis or other pathologies. His blood cultures and his urine culture and sensitivity were both found to be positive for ESBL-producing *E. coli* that was sensitive to ertapenem, indicating that the patient had developed urosepsis and bacteremia, and he was transitioned from piperacillin-tazobactam to IV ertapenem. On day three of admission, his renal function panel was notable for hyperphosphatemia, which continued to worsen by day five; his creatinine remained significantly elevated (Table 1). Clinically, the patient remained hemodynamically stable with no signs of septic shock.

**Figure 1:**
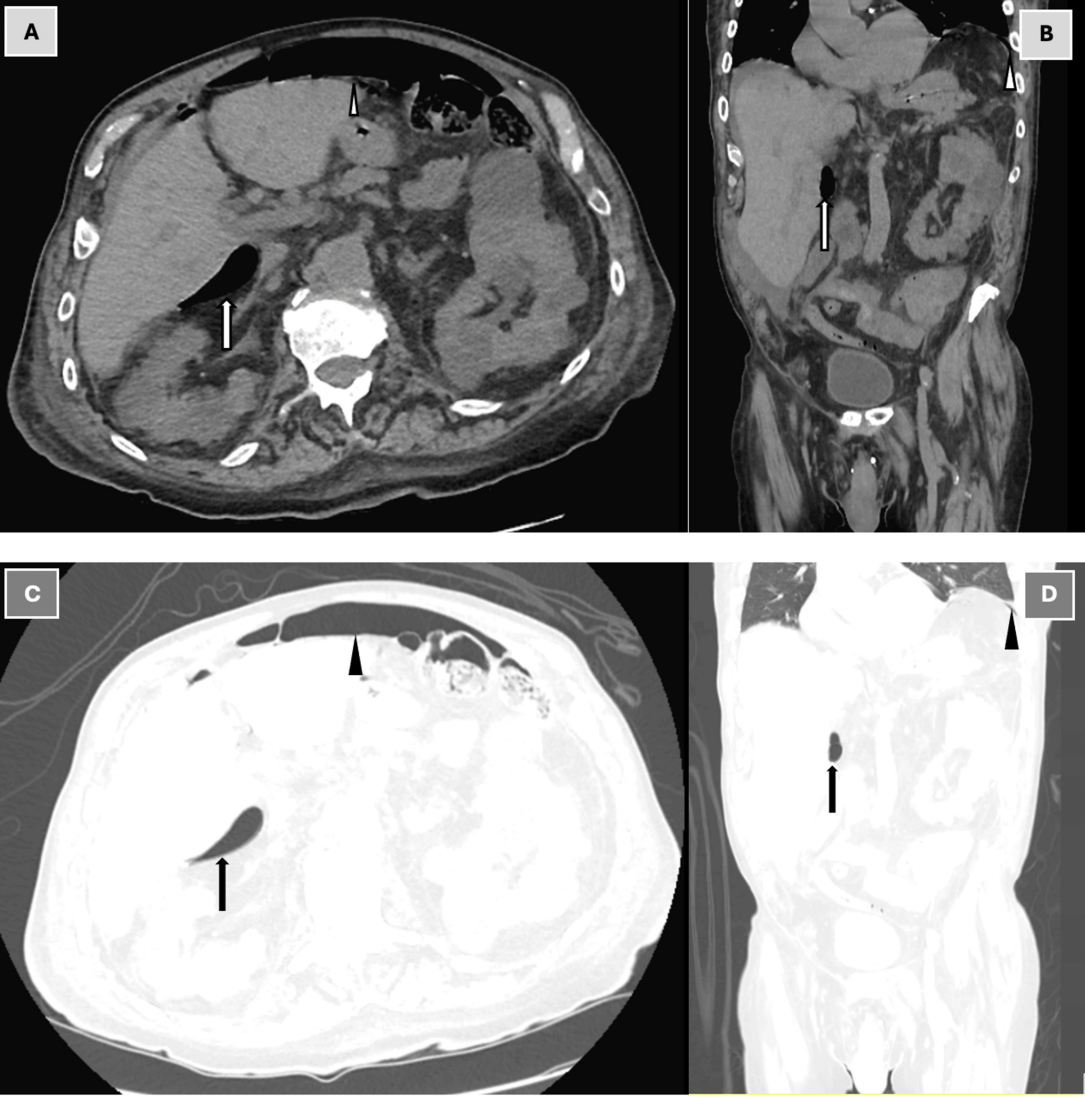
CT of the abdomen and pelvis without intravenous contrast: axial view in soft tissue window (A), coronal view in soft tissue window (B), axial view in lung window (C), and coronal view in lung window (D). CT scan of abdomen and pelvis without contrast showing findings of pneumoperitoneum (triangles) with periduodenal free air in the lesser sac (arrows), suggestive of a perforated duodenal ulcer.

On day five of his admission, the patient reported diffuse crampy abdominal pain for the past two and a half days and new-onset abdominal distension. He was not in active distress and reported that he had been having nonbloody bowel movements and passing of flatus. He denied nausea, vomiting, diarrhea, constipation, or any history of abdominal pain. The patient reported that his last colonoscopy was five years ago, which was negative aside from polyps, but he could not recall any prior upper endoscopy. Physical examination showed a distended abdomen with generalized tenderness, most severe in the right lower quadrant, with no rebound tenderness. Repeat laboratory tests showed a further elevation in his WBC count to 19.6 K/µL and neutrophil percentage to 93.7, along with newly elevated pancreatic enzymes with a lipase of 278 U/L and amylase of 327 U/L (Table 1). A kidney, ureter, and bladder scan was performed that showed a normal bowel pattern, but a repeat CT of the abdomen and pelvis without contrast demonstrated new findings of pneumoperitoneum with periduodenal free air in the lesser sac, ascites, small bilateral pleural effusions, and atelectatic changes in the lower lobe with concern for duodenal ulcer perforation (Figure 1). The general surgery team was consulted, and the patient was immediately taken into the operating room for an exploratory laparotomy under general anesthesia. During the procedure, a perforated anterior duodenal ulcer was found just distal to the pylorus with gross contamination of the peritoneum, and a sample of this peritoneal fluid was collected for culture and sensitivity testing before the fluid was washed out. The ulcer was debrided to healthy bleeding edges and was repaired using a modified Graham patch. For postoperative support, a gastrostomy tube was placed in Stamm fashion for gastric decompression, a jejunostomy tube was placed in Witzel fashion for early enteral nutrition, and a nasogastric tube was used for temporary postoperative decompression. The patient was continued on ertapenem, and an empirical anti-*H. pylori* treatment was initiated but promptly discontinued following negative testing.

Postoperatively, the patient was admitted to the surgical intensive care unit, and his renal function remained poor but clinically stable. He was made NPO for 48 hours to allow his tube sites to heal, which prevented the administration of phosphate binders for his hyperphosphatemia. The patient reported great relief from his previous abdominal pain, and his incisional wounds were intact with no signs of erythema, edema, or purulence. Over the next week, the patient was able to begin trickle feeds before being advanced to clear liquid, full liquid, and, finally, regular diet without complications. Micafungin was discontinued after a week with no signs of fungal infection. Given the patient’s continued hyperphosphatemia and uremia postoperatively (Table 1), on postoperative day three, he began to receive hemodialysis for three consecutive days and then began three-times-weekly dialysis that continued until his uremia and hyperphosphatemia had resolved. He experienced no complications from the repair of his duodenal ulcer perforation.

## Discussion

In the case of our patient, his perforated duodenal ulcer likely stemmed from idiopathic PUD, given his negative stool test for *H. pylori* infection as well as his lack of NSAID or aspirin use, and there was no evidence of other less common causes of PUD, such as vasculitis or Zollinger-Ellison syndrome. However, his negative stool antigen testing is limited by reduced sensitivity in the context of recent broad-spectrum antibiotic exposure; therefore, *H. pylori* infection could not be definitively excluded. However, in the absence of NSAID or aspirin use and with no evidence of other less common causes of PUD, this patient’s ulcer was most likely idiopathic, with its acute presentation exacerbated by the physiologic stress of AKI, potentially as a direct effect of his hyperuremia and anion gap metabolic acidosis, as well as the underlying physiologic stress on the body in AKI.

Patients with uremia, such as secondary to AKI, CKD, or end-stage renal disease (ESRD), have been shown to have a higher incidence of PUD, potentially stemming from increased gastrin levels or alterations in gastric acid secretion [3]. This potential damage from uremia in AKI, such as in the case of our patient who presented with a BUN of 140 mg/dL, could have caused the worsening of a preexisting peptic ulcer or incited the formation of an ulcer in the duodenum that subsequently perforated. Importantly, in the GRACE study involving 1,874 patients analyzing 30-day morbidity and mortality in adults who underwent surgical repair of perforated peptic ulcers, AKI was found to be a key factor associated with 30-day morbidity and mortality with an odds ratio of 7.9 [4]. This increased risk illustrates that, in addition to being a potential contributor to our patient’s duodenal ulcer perforation, his kidney injury was also a potent complicating factor in his recovery and likely significantly contributed to his risk of morbidity or mortality.

Additionally, our patient was under severe physiologic stress during his critical illness, as evidenced by his signs of severe dehydration, elevated WBC count, and anion gap metabolic acidosis on admission and in the five days before his duodenal ulcer perforation. Systemic stress in a critically ill patient contributes to the development of a stress-induced peptic ulcer, which has risk factors that include but are not limited to sepsis, metabolic acidosis, and AKI; however, this would be a relatively atypical presentation of a stress-induced ulcer, given that these ulcers typically occur in the fundus or body of the stomach rather than the duodenum and usually present with signs of upper gastrointestinal blood loss, including hematemesis, melena, anemia, hypotension, or shock, all of which our patient lacked during his hospital course [5]. Despite this being atypical presentation for a perforated stress-induced ulcer without signs of hemorrhage, our patient had the risk factors for the development of a stress-induced ulcer, and it is difficult to rule out his pathology involving some level of stress-induced mucosal damage, which is a common finding in 75-100% of critically ill patients within 24 hours of admission to an intensive care unit [6]. As a result, stress ulcer prophylaxis is recommended in critically ill patients with risk factors such as sepsis, AKI, or mechanical ventilation. Although primarily aimed at preventing hemorrhage, these measures may also reduce the risk of other serious complications, including perforation [6]. The often insidious nature of PUD, particularly idiopathic PUD, which may lack typical alarm symptoms until a catastrophic event such as perforation, poses a significant diagnostic challenge. This is further compounded in critically ill, elderly patients with multiple comorbidities, such as ours, where new or vague symptoms, such as diffuse abdominal pain, can be easily attributed to various ongoing processes or masked by altered mental status. Highlighting these distinctions helps contextualize this patient’s presentation as an atypical case, where both stress-related and idiopathic factors may have contributed to the duodenal ulcer perforation

Taking into account the wide-ranging systemic effects of AKI impacting every organ system via uremia, anion gap metabolic acidosis, hyponatremia, hyperphosphatemia, and even evidence in the literature of “AKI-induced distant organ crosstalk” leading to multiorgan dysfunction, it is critically important to maintain a low threshold for suspicion of acute extra-renal pathologies in these patients [7]. This vigilance in AKI patients should include a low threshold for investigating even subtle new abdominal signs, unexplained tachycardia, or worsening leukocytosis, as typical PUD symptoms may be masked, atypical, or absent. As our patient’s case illustrates, these effects can be atypical, and close monitoring, a thorough physical examination, and a wide differential diagnosis can all increase the speed with which such cases are detected and help reduce patient morbidity and mortality. However, as a single case report, the conclusions drawn have inherent limitations in generalizability. While the clinical picture strongly suggests a multifactorial etiology for the perforated ulcer, combining an underlying idiopathic PUD with exacerbation from uremia and physiological stress, the precise contribution of each factor and the exact timing of ulcer development before perforation could not be definitively established without prior endoscopic evaluation.

## Conclusions

This case emphasizes the complex interplay between AKI, systemic physiologic stress, and gastrointestinal pathology, particularly highlighting how uremia, metabolic acidosis, sepsis, and critical illness can exacerbate or contribute to peptic ulcer formation, leading to life-threatening complications such as duodenal ulcer perforation. While this patient’s presentation was atypical for a stress-induced ulcer, given its duodenal location and absence of hemorrhage, stress-related mucosal injury may have contributed alongside idiopathic PUD. Clinicians must remain vigilant for atypical presentations of extra-renal complications in patients with severe AKI, as early recognition and prophylactic measures targeting stress-induced ulcers can reduce morbidity and mortality. Close clinical monitoring, a comprehensive physical examination, and an expanded differential diagnosis remain essential to promptly identify and manage these complications.
